# PARP inhibitors in breast and ovarian cancer with BRCA mutations: a meta-analysis of survival

**DOI:** 10.18632/aging.202724

**Published:** 2021-03-11

**Authors:** Fengping Shao, Yaoyun Duan, Yunhe Zhao, Yinguang Li, Jun Liu, Cai Zhang, Shanyang He

**Affiliations:** 1 Department of Obstetrics and Gynecology, The First Affiliated Hospital, Sun Yat-Sen University, Guangzhou 510080, Guangdong, China; 2 Institute of Pediatrics, Guangzhou Women and Children’s Medical Center, Guangzhou Medical University, Guangzhou 510623, Guangdong, China; 3 Department of Gynecology and Obstetrics, Guangdong Provincial People’s Hospital, Guangdong Academy of Medical Sciences, Guangzhou 510080, Guangdong, China

**Keywords:** PARP inhibitor, BRCA mutation, breast cancer, ovarian cancer, efficacy

## Abstract

Objective: To evaluate the efficacy of poly ADP ribose polymerase (PARP) inhibitors (PARPis) in breast and ovarian cancer with *BRCA* (BReast CAncer susceptibility gene) mutation (BRCAm).

Methods: We conducted a meta-analysis of randomized controlled, phase II or III trials by searching of electronic databases from inception to September 1, 2020. The efficacy of PARPis measured by hazard ratios (HRs) and 95% confidence intervals (95% CIs) for progression free survival (PFS) and overall survival (OS) of patients.

Results: By addition of PARPis to conventional therapy, breast or ovarian cancer patients carrying BRCAm significantly benefited PFS (breast cancer: HR 0.64, 95% CI=0.55-0.75, P<0.001; ovarian cancer: HR 0.33, 95% CI=0.27-0.42, P<0.001), but OS of patients did not increase significantly in these two cancer types (breast cancer: HR 0.87, 95% CI=0.76-1.01, P=0.065; ovarian cancer: HR 0.78, 95% CI=0.61-1.01, P=0.058). For ovarian cancer patients carrying BRCAm, the use of therapy with PARPis yielded longer PFS at the stage of newly diagnosed than the stage of recurrence (22.5 months vs 9.6 months).

Conclusion: PARPis were beneficial to all with BRCAm, but they were "most" beneficial to the ovarian cancer subset when administered early after diagnosis, rather than after recurrence.

## INTRODUCTION

Cancer is the leading cause of death in the world and has become a major public health problem that has persisted worldwide, for a long time. Whether in developed or developing countries, many cancer-related deaths occur every day. For example, based on human epidemiology data, 4,950 people die of cancer every day in the USA [1], this is even worse in China, where over 7,500 people die of cancer, daily [2]. Obviously, all countries will face great challenges in dealing with the huge and increasing burden of cancer at present and in the foreseeable future. To overcome these challenges, it is necessary to increase investment in basic and clinical research to further promote treatment options, which will undoubtedly accelerate the progress of fighting cancer. Patients suffering with cancer still face the challenge of high recurrence rate after surgery and the toxicity of conventional chemotherapy, so safer and more effective treatment schemes are needed.

Targeted molecular therapy holds great promise for the treatment of cancer and represents a revolutionary breakthrough in personalized medicine. Among breast cancer and ovarian cancer patients with *BRCA* mutations, targeted therapy has always been a hot topic. In this regard, poly ADP ribose polymerase (PARP) inhibitor (PARPi), represent a novel cancer therapy targeting PARP, which have already achieved noteworthy therapeutic effects on cancer. Greater potency might be achieved by inhibiting PARP, because PARPi could sensitize cancer cells to conventional treatments including multiple chemotherapy or radiotherapy that cause DNA damage [3]. Using the genetic concept of synthetic lethality [3, 4], PARP inhibitors are designed to target cancers harbouring specific DNA-repair defects, including those arising in carriers of *BRCA1* or *BRCA2* mutations [5]. More promisingly, tumors exhibiting BRCAness are sensitive to PARPis, and the concept of BRCAness can be described as a defect in DNA damage response by homologous recombination repair, regardless of the presence of germline *BRCA1* or *BRCA2* mutations.

Excitingly, numerous studies have demonstrated that PARPis achieve excellent anti-tumor efficacy as monotherapy or combination therapy with conventional treatments in various cancers patients, especially in breast cancer and ovarian cancer. Based on the results of clinical trials, the ASCO guidelines recommended PARP inhibitors for the treatment of ovarian cancer, breast cancer, and pancreatic cancer with BRCA1 / 2 mutations [6–8].

Enhanced understanding of the efficacy of PARPis in ovarian and breast cancer patients carrying BRCAm will provide more accurate treatment information and can improve clinical decision-making. The objective of this study was to perform a meta-analysis to provide a clinical reference by comprehensively evaluating the OS and PFS of ovarian and breast cancer with *BRCA* mutations.

## RESULTS

### Literature search

**Figure 1:**
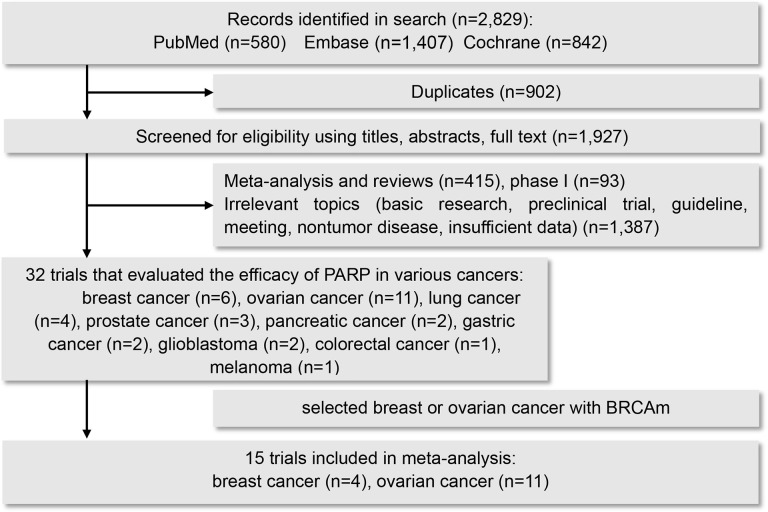
**Flow diagram for selection of studies.**

According to the search terms, a total of 2,829 related studies were identified from all searched databases. Because of duplications, 902 studies were removed. After eligibility screening of the titles, abstracts and full texts of the article, 1,895 studies were excluded because they have the characteristics of phase I clinical trial, basic research, preclinical trial, guideline, meeting, nontumor disease and insufficient data. After that, 32 trials related to evaluating the PFS or OS of PARPis in ovarian cancer, breast cancer, lung cancer, prostate cancer, pancreatic cancer, gastric cancer, glioblastoma, colorectal cancer and melanoma were retained. Finally, by analyzing breast and ovarian cancer with BRCA mutations, 15 randomized controlled trials were selected [9–27]. The flowchart of the trial selection process is shown in Figure 1.

### Study characteristics

The main characteristics of the eligible trials are showed in Table 1. 15 trials were involved in the analysis, including 11 trials concerning ovarian cancer, 4 trials concerning breast cancer. A total of 3,756 patients carrying BRCA mutations were included in the meta-analysis, of which breast cancer patients accounted for 40% and ovarian cancer patients for 60%. For ovarian cancer, more studies were performed to evaluate the efficacy of PARPis monotherapy as maintenance therapy, while for breast cancer, more studies were performed to evaluate the efficacy of combination of PARPis and conventional chemotherapy. Compared with the other PARPis, olaparib were mostly concerned about in these included clinical trials.

**Table 1:** Characteristics of the eligible trials in the meta-analysis.

**Cancer type**	**Trial, year**	**Phase**	**Therapeutic regimen**		**All patients** **(Exp/Con)**	**Cancer stage**	**Median PFS (Exp/Con)**
			**PARP inhibitor**	**Control**			
Ovarian cancer	Kaye 2012	II	Olaparib	Pegylated liposomal doxorubicin	64/33	Recurrent ovarian cancer	6.5 or 8.8/7.1 months
	Ledermann 2012,2014,2016	II	Olaparib	Placebo	136/129	Relapsed high-grade serous ovarian cancer	11.2/4.3 months
	Oza 2014	II	Olaparib plus chemotherapy, then olaparib	Chemotherapy then no further treatment	81/81	Recurrent high-grade serous ovarian cancer	not reported/9.7months
	Mirza 2016	III	Niraparib	Placebo	372/181	Recurrent high grade serous ovarian cancer	21.0/5.5 months
	Pujade-Lauraine 2017	III	Olaparib	Placebo	196/99	Relapsed high-grade ovarian cancer	19.1/5.5 months
	Coleman 2017	III	Rucaparib	Placebo	375/189	Recurrent high-grade ovarian carcinoma	16.6/5.4 months
	Moore 2018	III	Olaparib	Placebo	260/131	Newly diagnosed high-grade ovarian, primary peritoneal, or fallopian tube carcinoma	49.9/13.8 months
	Ray-Coquard 2019	III	Olaparib plus bevacizumab	Placebo plus bevacizumab	537/269	Newly diagnosed high-grade ovarian cancer, primary peritoneal cancer, or fallopian-tube cancer	37.2/21.7 months
	González-Martín 2019	III	Niraparib	Placebo	487/246	Newly diagnosed high-grade ovarian cancer, primary peritoneal cancer, or fallopian-tube cancer	22.1/10.9 months
	Coleman 2019	III	Veliparib plus carboplatin and paclitaxel then veliparib	Placebo plus carboplatin and paclitaxel then placebo	382/375	Newly diagnosed high-grade ovarian, fallopian tube, or primary peritoneal carcinoma	34.7/22.0 months
	Penson 2020	III	Olaparib	Single-agent nonplatinum chemotherapy	178/88	Relapsed high-grade ovarian cancer	13.2/8.5 months
Breast cancer	Robson 2017,2019	III	Olaparib	Standard therapy (capecitabine, eribulin, or vinorelbine)	205/97	Metastatic breast cancer	7.0/4.2 months
	Han 2018	II	Veliparib plus carboplatin /paclitaxel	Placebo plus carboplatin /paclitaxel	95/98	Recurrent/metastatic breast cancer	14.1/12.3 months
	Litton 2018, 2020	III	Talazoparib	Standard therapy (capecitabine, eribulin, or vinorelbine	287/144	Advanced Breast Cancer	8.6/5.6 months
	Diéras 2020	III	Olaparib	Placebo plus carboplatin and paclitaxel	337/172	Advanced Breast Cancer	14.5/12.6 months

### Efficacy of PARPis in breast or ovarian cancer with BRCAm, BRCA1m, BRCA2m

**Figure 2:**
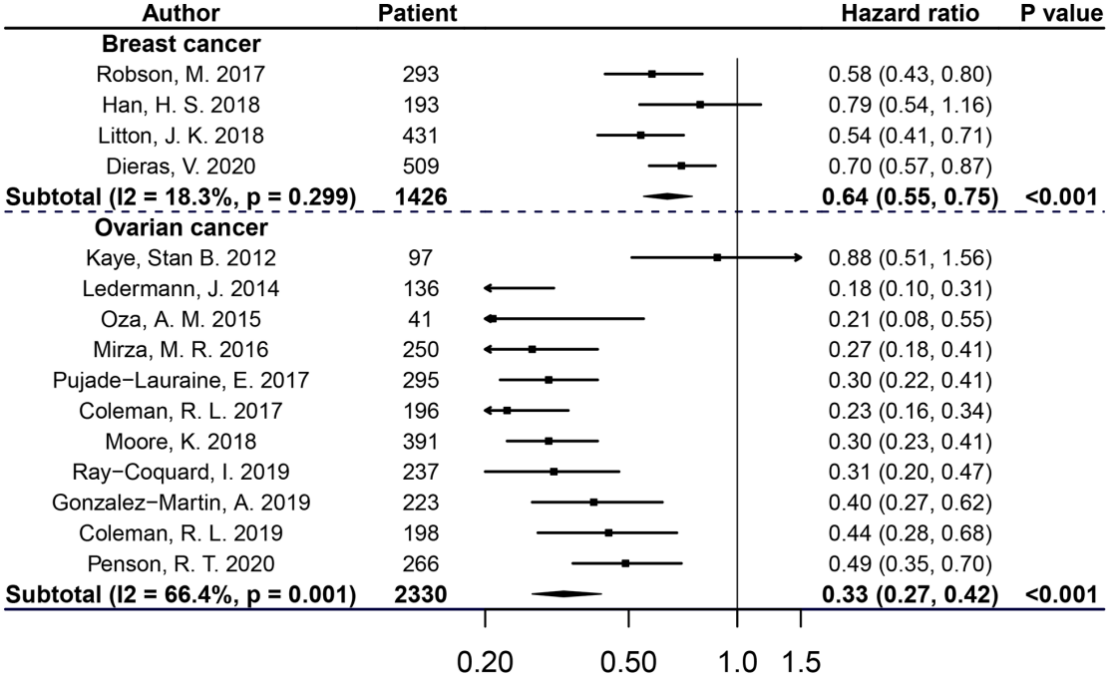
**PFS of breast or ovarian cancer patients with BRCAm treated with PARPis.**

**Figure 3:**
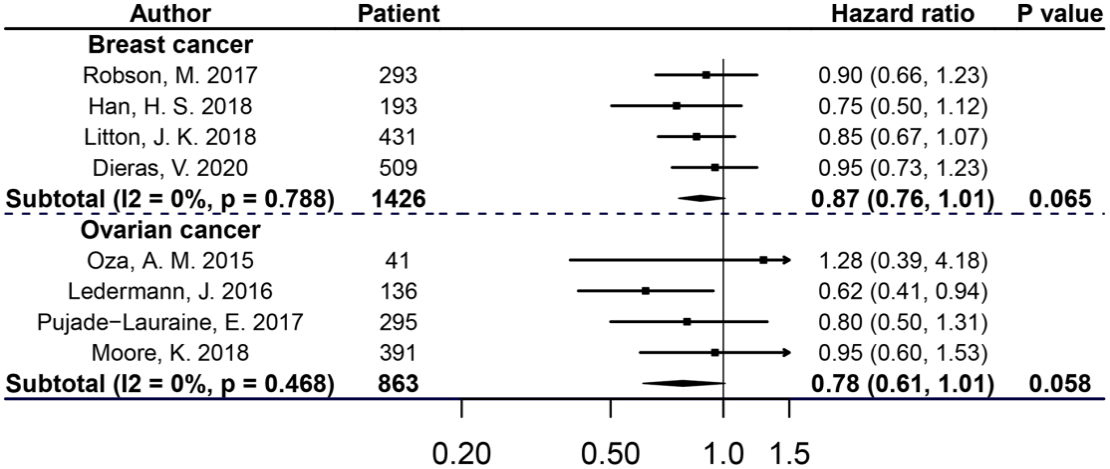
**OS of breast or ovarian cancer patients with BRCAm treated with PARPis.**

Significantly, patients with breast or ovarian cancer in PARPis treatment groups had a considerable advantage in PFS compared with control groups (for breast cancer, BRCAm: HR 0.64, 95% CI=0.55-0.75, P<0.001; BRCA1m: HR 0.64, 95% CI=0.53-0.78, P<0.001; BRCA2m:HR 0.62, 95% CI=0.51-0.76, P<0.001; for ovarian cancer, BRCAm: HR 0.33, 95% CI=0.27-0.42, P<0.001; BRCA1m: HR 0.38, 95% CI=0.29-0.48, P<0.001; BRCA2m: HR 0.24, 95% CI=0.10-0.59, P=0.002). However, compared with the control groups, PARPis did not improve OS in breast cancer or ovarian cancer patients (for breast cancer, BRCAm: HR 0.87, 95% CI=0.76-1.01, P=0.065; for ovarian cancer, BRCAm: HR 0.78, 95% CI=0.61-1.01, P=0.058). For breast cancer, no substantial heterogeneity was observed in subgroups of BRCAm, BRCA1m or BRCA2m cancers when assessing both PFS and OS; for ovarian cancer, heterogeneity exited in subgroups of BRCAm and BRCAm1 cancers when assessing PFS, but not in the other subgroups when assessing PFS or OS (see Figures 2, 3 and Supplementary Figures 1, 2).

### The efficacy of each PARPi in BRCAm cancer by pooling data from breast and ovarian cancer patients

**Figure 4:**
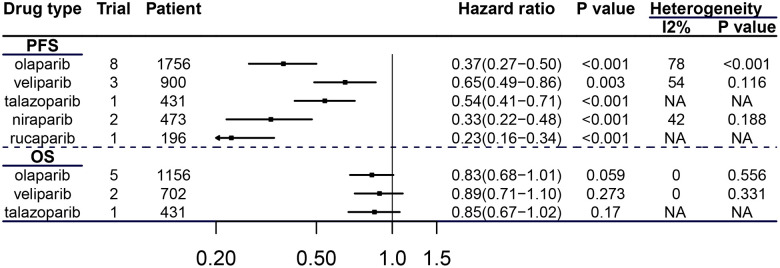
**Efficacy of each PARPi in breast and ovarian cancer patients with BRCAm.**

Compared with the control groups, all PARPis included in the analysis were statistically significant in improving PFS of patients with BRCAm cancers. Compared olaparib group with control group, HRs for PFS and OS of cancer patients with BRCAm were 0.37 (95% CI=0.27-0.50, P<0.001), 0.83 (95% CI=0.68-1.01, P=0.059), respectively (see Figure 4).

### The efficacy of PARP inhibitors used with different intervention methods

**Figure 5:**
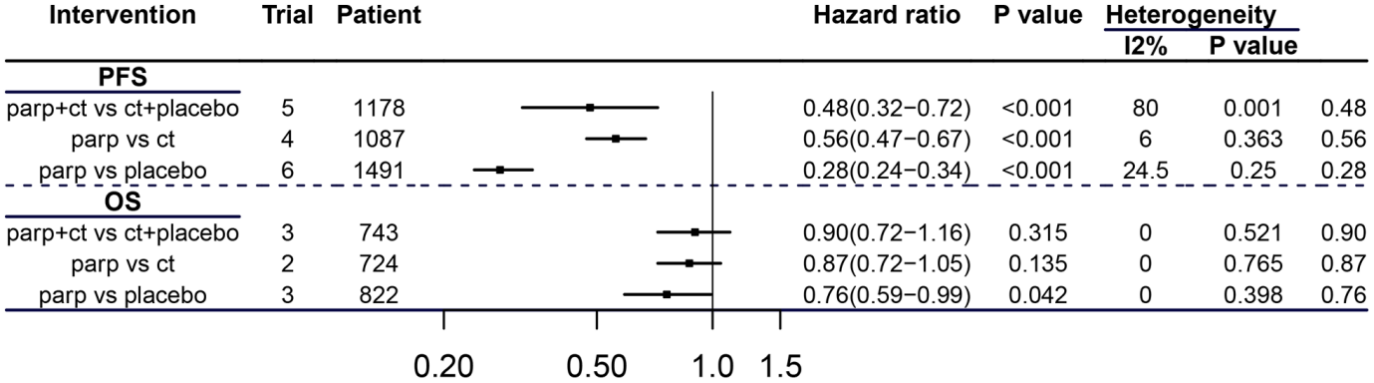
**Efficacy of PARPis with different interventions in breast and ovarian cancer patients with BRCAm.** CT = chemotherapy.

Compared with the control groups, PAPRis with different intervention methods were all statistically significant beneficial for PFS of patients carrying BRCAm by integrating data from these two cancer types. (parp+ct vs ct+placebo: HR 0.48, 95% CI=0.32-0.72, P<0.001; parp vs ct: HR 0.56, 95% CI=0.47-0.67, P<0.001; parp vs placebo: HR 0.28, 95% CI=0.24-0.34, P<0.001); for subgroup of “parp vs placebo” PARPis had a statistically significant advantage over placebo for OS of patients in these subgroup (see Figure 5) (HR 0.76, 95% CI=0.76-0.99, P=0.042).

### Efficacy of PARPis in patients with *BRCA* mutant ovarian cancer at different stages of development

In patients with recurrent ovarian cancer, median PFS was 15.1 months in the PARPis treatment group versus 5.5 months in the control groups, yielding a PFS benefit of 9.6 months; remarkably, in patients with newly diagnosed advanced ovarian cancer, it was 36 months versus 13.5 months, prominently yielding a PFS benefit of 22.5 months (see Table 2). Therefore, the patients of BRCAm ovarian cancer can get better survival when PARPis are administered earlier in cancer progression.

**Table 2:** Median PFS of patient with BRCA mutant ovarian cancer treated with PARPis.

**Stage of cancer**	**Trial**	**Median PFS months(exp)**	**Median PFS months (con)**	**N patients (exp)**	**N patients (con)**
Recurrence	Kaye 2012	6.5/8.8	7.1	32/32	33
	Ledermann2012,2014,2016	11.2	4.3	74	62
	Oza 2014	NA	9.7	20	21
	Mirza 2016	21	5.5	138	65
	Pujade-Lauraine 2017	19.1	5.5	196	99
	Coleman 2017	16.6	5.4	130	66
	Penson 2020	13.2	8.5	178	88
**Pooled patient-level data**		**15.1**	**5.5**	**800**	**434**
Newly diagnosed	Moore 2018	49.9	13.8	260	131
	Ray-Coquard 2019	37.2	21.7	157	80
	González-Martín 2019	22.1	10.9	152	71
	Coleman 2019	34.7	22	108	92
**Pooled patient-level data**		**36**	**13.5**	**677**	**374**

NA=not available.

### Publication bias

As visually assessed, substantial asymmetry was not identified in the Begg funnel plot (see Supplementary Figure 3). Moreover, no significant publication bias was found by the Begg rank correlation test and Egger linear regression test.

## DISCUSSION

Rapid death is the inevitable outcome of patients with advanced or metastatic breast and ovarian cancer. It is urgent that an effective solution emerges to manage patients of advanced or metastatic breast and ovarian cancer.

Multiple meta-analysis of patients with ovarian cancer or breast cancer had proved that addition of PARPis to therapy was beneficial [28–31]. Moreover, Gu, L., et al. included 12 clinical trials containing six types of cancer patients for meta-analysis, and then concluded that with the acceptable and controllable toxicity profile, PARPis improved survival of cancer patients, and were more beneficial to ovarian cancer patients with *BRCA*m [32].

In order to evaluate PARPis in-depth and in detail, this meta-analysis comprehensively focused on evaluating the efficacy of each PARPi, with respect to different intervention strategies in patients with BRCAm-positive breast and ovarian cancer, and also differences in the therapeutic effectiveness of PARPis when administered at the time of relapse versus at the time of ovarian cancer diagnosis. In a subgroup of BRCAm cancers, our pooled analysis showed that compared with the control, the PARPis treatment group showed a statistically significant reduction in disease risk progression of patients with breast (36%) or ovarian (67%) cancer. From the perspective of OS data from meta-analysis, more RCTs are needed to confirm whether PARPis effectively improve the OS of *BRCA* mutant patients with breast or ovarian cancer.

For ovarian cancer, despite standard therapy which includes cytoreductive surgery and conventional chemotherapy, about 70% of patients with newly diagnosed advanced ovarian cancer will face to relapse within the subsequent 3 years [33]. Promisingly, Ibrahim, E.M., et al. demonstrated that in newly diagnosed patients with advanced high-grade ovarian cancer, PARPis significantly decreased the risk of PFS by 46% when compared with placebo [34]. As first-line maintenance therapy, PARPis greatly benefit PFS of patients with newly diagnosed advanced ovarian cancer [35]. Consistent with these results, our study found that the addition of PARPis to standard therapy at the beginning of diagnosis for advanced ovarian cancer patients with BRCAm lead to an additional PFS benefit of 12.9 months, when compared at the time of relapse.

### Use PARPis in patients of cancer with homologous recombination deficiency (HRD)

Importantly, by using novel biomarkers of homologous recombination repair deficiency, the benefits of PARPis were extended to wider populations of patients beyond breast or ovarian cancer, even beyond *BRCA* mutant cancer [36]. For other cancers, in patients with BRCAm and metastatic pancreatic or prostate cancer, olaparid group had a better PFS than control group [37, 38]. Furthermore, for patients with HRD, PARPi used in the populations of *ERCC1* or *BRCA* deficient cancers, might potentiate their therapeutic effects by regulating the signal pathway related to antitumor immunity [39, 40]. ATM (ataxia telangiectasia mutated) promoted survival by decreasing sensitivity to PARP inhibition and playing a role upstream of homologous recombination repair in the repair of certain types of double-strand breaks [41]. PARP inhibitors had a significant killing effect on many cancers with ATM deficient [42–45]. Results from a phases 3 trial enrolled prostate cancer patients with qualifying deleterious or suspected deleterious alterations in at least 1 of 15 prespecified genes in homologous recombination repair: *BRCA1, BRCA2, ATM, BRIP1, BARD1, CDK12, CHEK1, CHEK2, FANCL, PALB2, PPP2R2A, RAD51B, RAD51C, RAD51D,* and *RAD54L*, indicated that olaparib improved progression-free survival when compared with enzalutamide or abiraterone [38]. However, because there is a lack of reliable clinical data to confirm PARPis are suitable for treating all types of cancer, patients should have genetic testing for defects in BRCA1/2 and other genes related to homologous recombination deficiency, before PARPis are safely applied [46].

### Strengths and limitations of study

Strengths: Firstly, 1,426 breast cancer patients and 2,300 ovarian cancer patients with BRCA mutations were included in this analysis. Secondly, through reasonable stratification and grouping, the therapeutic characteristics of PARPis in patients with breast or ovarian cancer carrying BRCAm, BRCA1m and BRCA2m were analyzed. Thirdly, after considering the pooled, satisfactory results of median PFS in newly diagnosed patients with *BRCA* mutant ovarian cancer, we recommended PARPis as the first-line maintenance therapy for BRCAm ovarian cancer.

Limitations: We acknowledge that this evidence-based medical report has some limitations, based on heterogeneity from cancer type, PARPi type and therapeutic schedule, which may reduce the accuracy of our results. Firstly, two cancer types were included in this study, and no further detailed analysis was made in pathological types in each cancer. Secondly, diversification existed in the phase of the treatment for patients, as well as the treatment options of PARPis. Thirdly, data on the OS of several studies was not mature enough or published. Fourthly, although stratification analysis was conducted, a few trials were included in some subgroup analysis.

## CONCLUSIONS

In this meta-analysis, application of therapy with PARPis provided a substantial PFS benefit among breast and ovarian cancer with BRCA mutations; and among patients with BRCAm ovarian cancer, PARPis provided longer PFS benefit at the stage of newly diagnosed than at the stage of recurrence.

## MATERIALS AND METHODS

We performed a meta-analysis of PARPi efficacy in *BRCA* mutant cancers according to the recommendations of the Cochrane Handbook and PRISMA statement guidelines [47].

### Search strategy

The search strategy and selection criteria are similar to the previous study we published [29]. We conducted a comprehensive systematic search of PubMed, Embase and Cochrane from inception to September 1, 2020 for all RCTs, and then checked the trial registration number and more relevant information in the https://www.clinicaltrials.gov/ and international clinical trials registry platform. For database searches we used the “parp OR poly adp ribose polymerase OR poly adenosine diphosphate ribose polymerase OR olaparib OR veliparib OR iniparib OR rucaparib OR niraparib OR talazoparib” as the search terms in all fields.

### Selection criteria

Exclusion criteria and inclusion criteria were prespecified and used in literature search and screening. To be eligible, the selected randomized controlled trials not only met the condition of researching the clinical efficacy of PARPis in patients, but also met the following conditions. Firstly, the population was patients with breast and ovarian cancers carrying *BRCA* mutations, irrespective of cancer stage or grade, surgery, recurrence, drug resistance, histology. Secondly, intervention: treated with PARPis (olaparib, veliparib, rucaparib, iniparib, talazoparib, niraparib) as monotherapy or combination with conventional chemotherapy or molecular target therapy regardless of dosage and duration. Thirdly, main outcome: the primary outcome was OS or PFS measured as HR. Studies were excluded if they were non-randomized control trials, phase I clinical trials, literature reviews and meta-analysis, case reports, retrospective or prospective observational cohort studies, basic science papers, commentaries, quality of life studies, and cost effectiveness analyses. In addition, we excluded those studies that did not explore PFS and OS or whose data have not yet been published. Moreover, updated and published follow-up data meeting the inclusion criteria which may have appeared in multiple articles or different publications were considered for one trial to analyze.

### Data extraction and risk of bias assessment

The key purpose of this meta-analysis was to evaluate the efficacy of PARPis measured by HRs of OS or PFS. Using a pilot-tested data extraction sheet, two investigators independently reviewed the eligible literature, and extracted the data including: cancer type and clinical stage or grade, first author, year of publication, phase of clinical trial, number of patients enrolled, intervention method, hazard ratios (HR) and their 95% confidence intervals (CIs) for OS and PFS stratified by *BRCA* status. The risk of bias was evaluated by using the Cochrane Risk of Bias Tool that consists of random sequence generation; allocation concealment; blinding of participants and personnel to the study protocol; blinding of outcome assessment; incomplete outcome data; and selective reporting [48]. The risk of bias was divided into three different levels: high, low, or unclear. Two investigators completed the review independently and in the event of any differences, resolved them through discussion and consultation.

### Statistical analysis

The primary objectives of the meta-analysis were OS and PFS in breast and ovarian cancers patients with BRCAm. Subgroup analysis was conducted to explore the efficacy of PARPis in breast or ovarian cancer by stratification BRCA1m or BRCA2m, and also to evaluate the efficacy of each PARPi and different intervention methods by integrating data from these two cancer types. Cochrane’s Q-test and I2 statistics were used to assess heterogeneity across the different studies. P ≤ 0.10 or I^2^ ≥ 50% indicated significant heterogeneity. The random-effect model was used to increase reliability because of the obvious heterogeneity attributed to differences by cancer type, PARPi type and therapeutic schedule in this meta-analysis [32]. Therefore, it was necessary to perform subgroup analysis to reduce heterogeneity and improve reliability. Potential publication bias was assessed by the Begg’s and Egger’s test [49, 50]. P < 0.05 was refer to indicate statistical significance. All analysis was carried out using Stata version 15.1 (StataCorp, College Station, TX, USA).

### Data availability statement

All data relevant to the study are included in the article or uploaded as supplementary information. All data generated or analysed during this study are included in this published article (and its supplementary information files).

## Supplementary Material

Supplementary Figures
