# Supplementary material for: PARP inhibitors in breast and ovarian cancer with BRCA mutations: a meta-analysis of survival
Source: Aging (Albany NY). 2021 Mar 11;13(6):8975–88. doi: 10.18632/aging.202724 (PMC8034970; doi:10.18632/aging.202724)
Supplement: Supplementary Figures [file aging-13-202724-s001.pdf]

SUPPLEMENTARY FIGURES

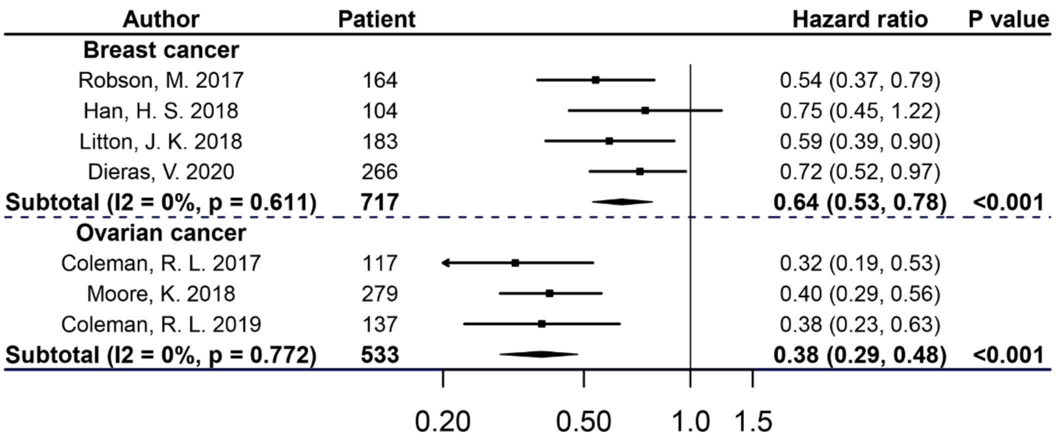

Supplementary Figure 1. PFS of breast or ovarian cancer patients with BRCAm1 treated with PARPis.

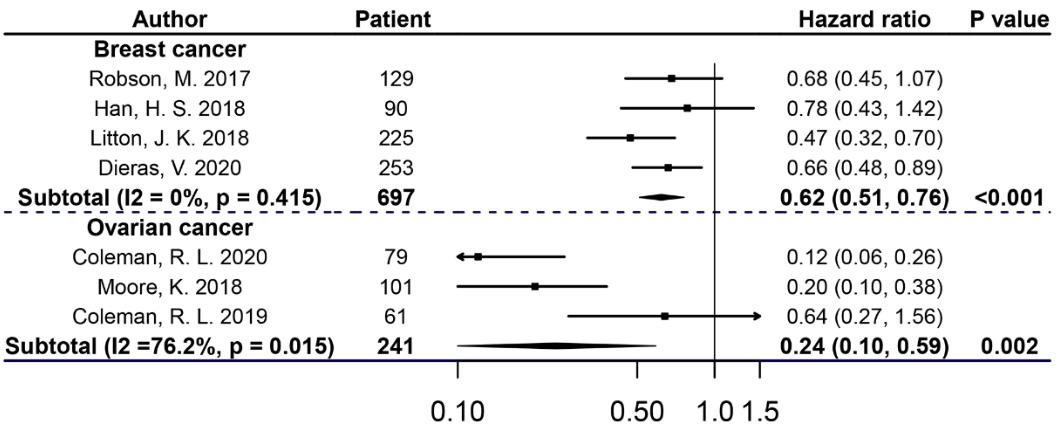

Supplementary Figure 2. PFS of breast or ovarian cancer patients with BRCAm2 treated with PARPis.

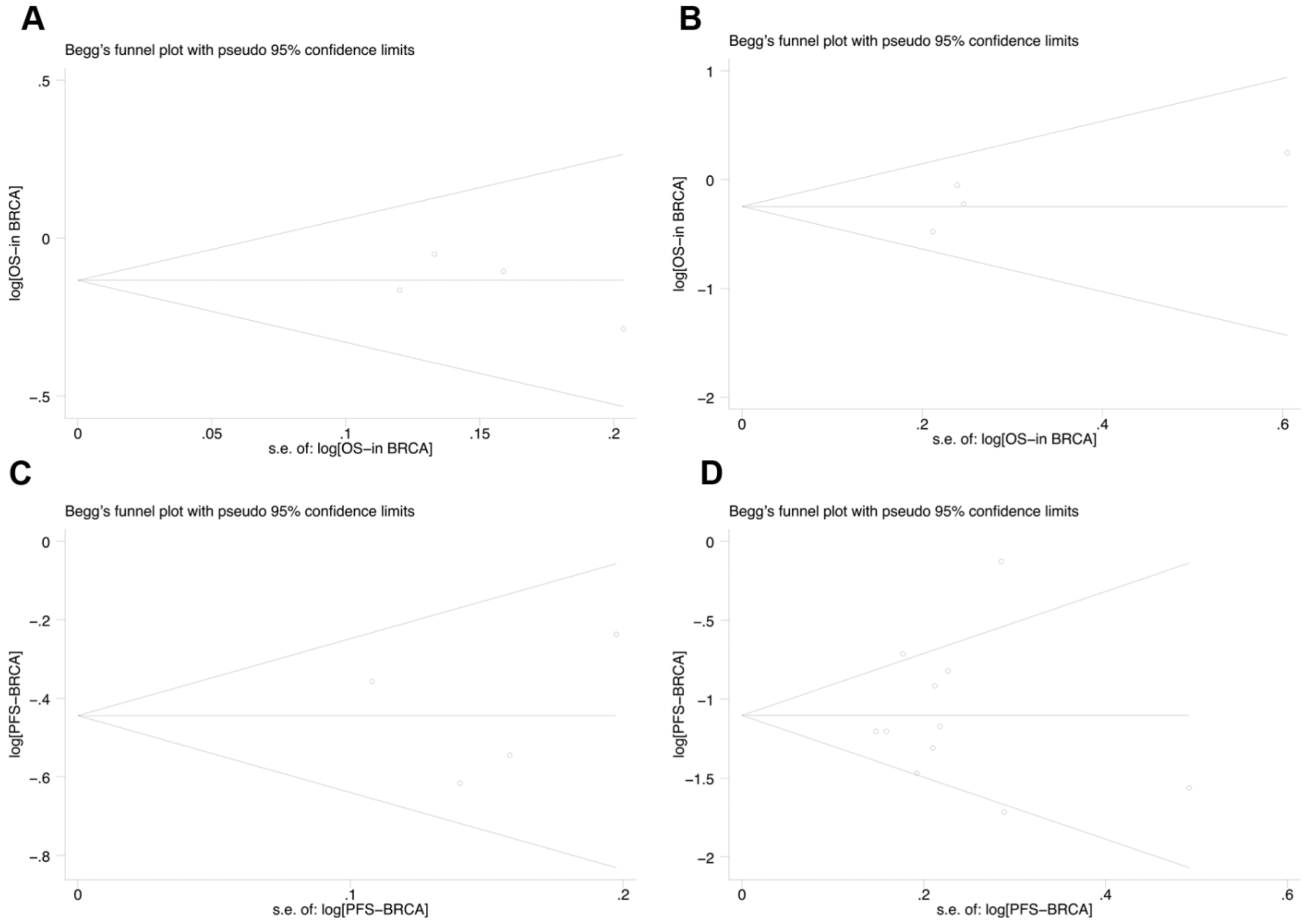

Supplementary Figure 3. Begg's funnel plot for publication bias test for survival of breast cancer group (**A**, **C**) and ovarian cancer group (**B**, **D**) with BRCAm.
